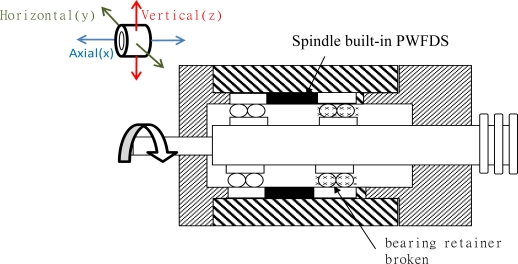# The Development of a Monitoring System Using a Wireless and Powerless Sensing Node Deployed Inside a Spindle

**DOI:** 10.3390/s120100024

**Published:** 2011-12-22

**Authors:** Liang-Cheng Chang, Da-Sheng Lee

**Affiliations:** 1 Graduate Institute of Mechanical and Electrical Engineering, National Taipei University of Technology, No. 1, Sec. 3 Zhongxiao E. Rd., Taipei City, 106, Taiwan; 2 Department of Energy and Refrigerating Air-Conditioning Engineering, National Taipei University of Technology, No. 1, Sec. 3 Zhongxiao E. Rd., Taipei City, 106, Taiwan; E-Mail: f11167@ntut.edu.tw

**Keywords:** monitoring system, wireless and powerless sensing node, signal-to-noise ratio, spindle

## Abstract

Installation of a Wireless and Powerless Sensing Node (WPSN) inside a spindle enables the direct transmission of monitoring signals through a metal case of a certain thickness instead of the traditional method of using connecting cables. Thus, the node can be conveniently installed inside motors to measure various operational parameters. This study extends this earlier finding by applying this advantage to the monitoring of spindle systems. After over 2 years of system observation and optimization, the system has been verified to be superior to traditional methods. The innovation of fault diagnosis in this study includes the unmatched assembly dimensions of the spindle system, the unbalanced system, and bearing damage. The results of the experiment demonstrate that the WPSN provides a desirable signal-to-noise ratio (SNR) in all three of the simulated faults, with the difference of SNR reaching a maximum of 8.6 dB. Following multiple repetitions of the three experiment types, 80% of the faults were diagnosed when the spindle revolved at 4,000 rpm, significantly higher than the 30% fault recognition rate of traditional methods. The experimental results of monitoring of the spindle production line indicated that monitoring using the WPSN encounters less interference from noise compared to that of traditional methods. Therefore, this study has successfully developed a prototype concept into a well-developed monitoring system, and the monitoring can be implemented in a spindle production line or real-time monitoring of machine tools.

## 1. Introduction

Using a monitoring system to assess the symptoms of mechanical systems prior to the occurrence of faults can effectively prevent sudden system shutdowns and any consequent impact on productivity [1]. A wide variety of monitoring methods target various sensors for machine tools, such as temperature sensors, displacement sensors, speedometers, and accelerometers [2]. The most commonly referenced indicator of damages to the bearing of the machine tool spindle is the vibration signal. The magnitude and frequency of vibration can be used to identify the cause of system failure, such as bearing damage, impeller failure, sealing failure, and mixed failure spectrum [3,4]. With advancements in industrial technology, increasingly diverse methods are developed to analyze the faults of machine tools, including identifying the fault cause of a single component even with multiple vibration sources [4]. Subsequently, additional methods can be used to analyze system faults, such as an artificial neural network, decision tree, and fuzzy fault diagnosis [5–8]. Apart from identifying the type of system failure, a number of studies have examined the identification of a fault cause in systems with a low SNR [9,10]. To diagnose and analyze the cause of faults in systems with a low SNR, a complex mathematical system must be applied; however, a low SNR still cannot eliminate the possibility of identification errors. This study is based on prior research by author Lee, titled *Wireless and Powerless Sensing Node System*, and published by Sensors in 2008 [11]. Lee’s system adopts wireless and powerless sensing node technology, and transmits signals across a metal case of a certain thickness. The sensor can be installed inside the core of the spindle to obtain vibration signals near the fault source. This study develops a Wireless and Powerless Monitoring System (WPMS) using the key technology of signal transmission across a metal case. The actual fault diagnosis occurs inside the spindle system. Using lengthy tests and adjustments, three faults were identified. When applied to an actual spindle production platform, the proposed system could improve the SNR of system monitoring, as well as the diagnosis rate of system faults.

**Figure 1.**
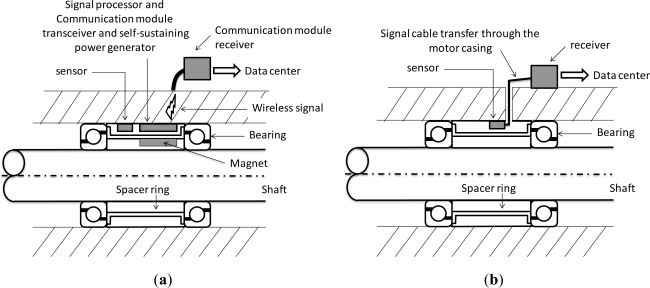
Schematic views of (**a**) the condition monitoring system with a wireless and powerless sensing node and (**b**) a traditional system.

This study applies a fault diagnosis system to the core of the spindle system to monitor the vibration frequency inside the spindle system, which differs from traditional systems where the metal case of the spindle system must be pierced to enable monitoring. Figure 1(a) shows the schematic view of the spindle monitoring system with a wireless and powerless sensing node; Figure 1(b) shows the installation of the traditional system of vibration monitoring. With the traditional monitoring method, a hole must be created in the metal case of the spindle, and special precautions adopted to prevent any damage to the signal cable and the power cable during assembly, because damage to the signal cable may lead to incorrect information regarding vibrations. The more sensor types used, the more complicated the wiring is. Therefore, this study proposes the innovative concept of placing the wireless and powerless sensing node in the spindle. In this approach the rotor includes a permanent magnet that drives the power-generating module of the wireless and powerless sensing node. The WPMS is placed at the spacer ring between the bearings to monitor the vibration signals of the bearing using the nearest location to the bearing. This fault diagnosis system monitors the operational status of the spindle without requiring power cables and signal cables. Distinct from traditional systems, the proposed WPMS conducts system monitoring without damaging the external structure of the spindle, which not only reduces the monitoring system costs and improves the fault identification rate, but also enables modularized production and reduces the complexities of assembly and maintenance.

## 2. The WPMS Applied to the Spindle System

Monitoring the operational status of a spindle using a WPMS is an innovative application. No previous cases using a similar approach have been found in academic research or industrial applications. Therefore, implementing the system on the spindle of machine tools presents a significant challenge. Based on discussions regarding the characteristics of wireless and powerless signal transmission in the article entitled WPSN, this study achieves greater stability and a higher identification rate following over two years of continuous research. The module specifications of the selected WPMS are given in the sections that follow.

### 2.1. Selection of the Sensors

The most commonly used indicator for monitoring the normal operations of a spindle system are the vibration signals. Abnormality in the operation of the spindle can be identified immediately through these vibration signals, whose magnitude and frequency vary for different faults. The sampling frequency of the vibration sensors imposes restrictions on the resolvable faults of the diagnosis system. Traditionally, piezoelectric accelerometers are used for fault diagnosis, which is extremely costly if an accelerometer must be installed on every spindle. Additionally, piezoelectric accelerometers are relatively large, which hinders their assembly on the core of the main spindle. This study selects the MEMS-type accelerometer. A comparison between the MEMS accelerometer and a traditional piezoelectric accelerometer is shown in Table 1. The monitoring range of the MEMS accelerometer is ±6 g, with a resolution scale of 1.22 mg, compliant to the 2.5 g maximum magnitude of fault vibration. The basis used to monitor the acceleration is differential capacitance. Acceleration displaces a silicon structure, changing the capacitance. Therefore, the MEMS accelerometer is an acceleration wafer with comparatively good linearity and suitable for long-term monitoring of the spindle operations. Unlike a traditional piezoelectric accelerometer, the MEMS accelerometer does not require the addition of an adjusted circuit, nor does it require an amplifier. Additionally, it consumes minimal energy and is compact in size, making it suitable for WPMS.

### 2.2. RF Communication Module

Regarding the transmission of vibration signals using a metal case with the WPMS, the most critical factor is preventing signal distortion. During wireless data transmission, the sampling time of the sensors is notably affected by the timing device and the processing speed of the wireless module. The ordinary wireless transfer rate is approximately 100 to 300 kbs, which is lower than that of a cable transfer. Additionally, the sampling time is subject to the processing capability and the size of storage. Ensuring a normal power supply to the wireless system is also a critical issue.

**Figure 2.**
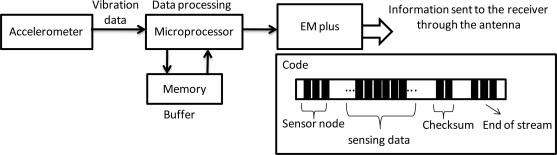
Schematic view of the signal processing and encoding of the wireless and powerless fault diagnosis node.

This study adopts a batch-type circuit design for the wireless and powerless fault diagnosis node, and saves the data of vibrations collected by the accelerometer into memory using the microprocessor. The microprocessor specifies that the sampling frequency of the accelerometer is 0.25 ms/time, and the data are transmitted in packets by the RF Chip every 4,000 entries. To avoid data loss during transmission, the sampling frequency of the accelerometer is not affected by the transmission rate of the RF Chip. The batch-type information processing procedure is shown in Figure 2. This method has a 1 to 5 s processing time during each packet transmission where the vibration data between the intervals is lost. However, the actual operation revealed that when a fault vibration occurs, the machine tool does not lose important data because of these 5 s required for data processing.

This data process is based on records from the accelerometer, then complete data is gathered and saved into memory. To avoid inappropriate data loss, this study formulated a code comprised of a sensor node, sensing data, checksum, and end of stream. The start and end of data reading is confirmed by the sensor node and the end of stream, respectively, and the checksum is examined to validate the accuracy of the data. In addition, both the transmission and the reception of data are demodulated using Manchester encoding. If data transmission fails, the system is able to repeat resend the previous data immediately. The checksum functionality is designed to prevent this circumstance from happening in any spindles. Therefore, a complete set of vibration data is returned when the machine tool operates under a batch-type hardware framework, which can be used to monitor whether the spindle system is experiencing a fault.

In the article titled *Wireless and Powerless Sensing Node System* it was reported that transmitting data across the 20 mm metal case using EM with the communication module results in a 5.3 dBm loss. This study discovers that the industrial RFID chip nRF905 can produce a maximum transmission power of 10 dBm, and the chip itself, which comprises a frequency synthesizer, power amplifier, crystal oscillator and modulator, works on two configurable frequencies, 430 MHz and 433 MHz. In addition, the data is demodulated upon reception. Manchester encoding and demodulation is automatically conducted during the transmission and reception. The encoding and demodulation also ensure the accuracy of the data transmission, and protect packets from distortion. Based on these considerations, the RFID chip nRF905 is adopted as the chip for data transmission in this study. The specifications of the chip are provided in Table 2.

### 2.3. The Design of the Embedded Power Generating Module

**Figure 3.**
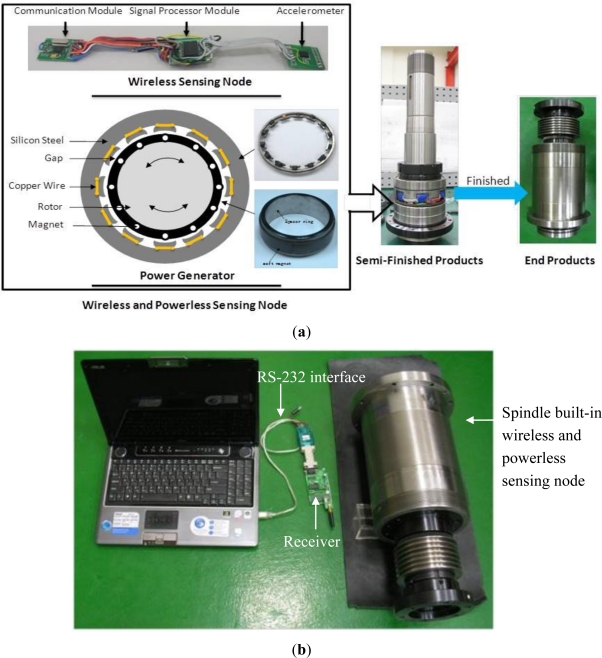
Fault diagnosis system (**a**) the wireless and powerless sensing node installed at the spindle system (**b**) the receptive module outside the spindle, which receives data from the wireless and powerless sensing node.

This study develops a power generating system to supply electricity to the wireless and powerless fault diagnosis module. A permanent magnet is installed on the main spindle, and when the spindle spins, the permanent magnet is driven to spin together. An induction coil is included in the stator, which generates induced potentials by alternating magnetic fields. The design is shown in Figure 3(a). The soft magnet on the rotor is permanent, made from rubber, and fixed to the internal spacer ring of the rotor by wrapping around the outside of the spacer ring of the spindle. The magnet has a remanent flux density (Br) of 254 mT, a maximum energy product (BH) of 11.1 kJ/m^3^, and an operating temperature range of −40 to 100 °C. Additionally, the permanent magnet has 12 poles, and the distance between the rotor and the stator is 2 mm. Through the circuit design of the rectifier and filter regulator, when the rotor spins at a rate of 1,000 to 6,000 rpm, a stable output voltage of 3.2 V and an output power of 1 to 5 W is generated. Because the WPMS consumes 0.12 W, the power generating module is capable of supplying sufficient voltage and power to the embedded system.

### 2.4. Integration of the Embedded System

To collect sensitive vibration signals, the accelerometer monitoring the vibration signals on the spindle should be placed near the shearing force of the bearing to determine whether the forces acting on the bearing are balanced, and whether the bearing is broken. Therefore, a WPMS with an accelerometer is installed between the two bearings inside the spindle. Regarding the wireless and powerless sensing node, because no wiring is required, the need for changes in the design of the spindle structure is reduced. Additionally, the modularized design simplifies the assembly process and a considerable amount of processing and labor costs can thus be reduced in spindle production.

The WPMS includes a wireless and powerless sensing node and a power generator. The wireless and powerless sensing node is an integrated system comprising a sensor, microcontroller, RF chip, and memory, and manages data using the bench mode, where the transception of data is conducted through the metal case of the spindle. The wireless and powerless sensing node and the power generator are installed at the spacer ring of the spindle, which is placed between the front and rear bearings of the spindle. The system components for assembling the semifinished product and the final product are shown in Figure 3(a). The blue parts in Figure 3(a) denote the wireless and powerless sensing node encapsulated by insulated packaging materials. The external receiver of the system is a receptive module comprising a RFID chip nRF905, microprocessor, and RS-232 conversion chip. The module receives the data sent by the WPMS, and transmits the data to the computer for data recording. The complete system architecture is shown in Figure 3(b).

## 3. Experiments

### 3.1. Transferring Data through the Spindle Metal Shaft

**Figure 4.**
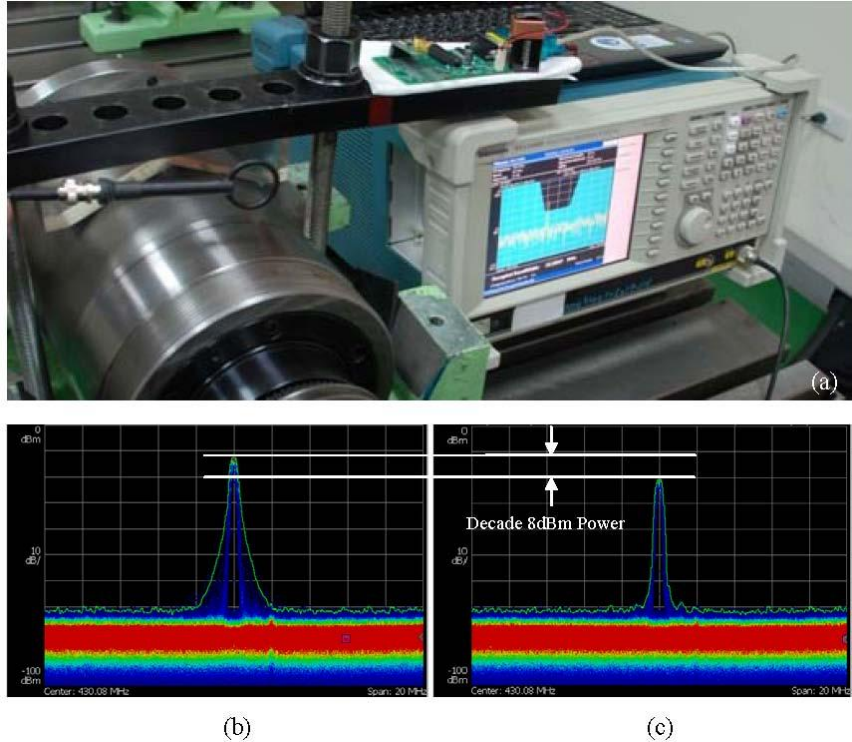
The results of the power attenuation of the signals transmitted through a metal case. (**a**) Architecture of the testing system. (**b**) The results measured at a distance of 20 mm in free space. (**c**) The results measured across a 20 mm thick metal case.

Because the WPMS transmits data across the metal case, the signal attenuation after passing through the case is extremely important. Considering the characteristics of wireless and powerless signal transmission through a metal case, this study tests the attenuation of the signals after penetrating the metal case. The Tektronix RSA3408B Real-Time Spectrum Analyzer is used to test the power of the electromagnetic wave. The structure of the experiment, and a comparison between the signal attenuation after passing through the case and the attenuation passing through air for the same distance, is shown in Figure 4(a).

The results of the experiment are shown in Figure 4(b,c). Figure 4(b) displays the power spectrum of the WPMS passing through air when measured at a distance of 20 mm, which reveals that the power of the signal is −12 dBm, and the general noise is −70 dBm. Figure 4(c) displays the power spectrum of the WPMS passing through the metal case, which reveals that the power of the signal after penetration is −20 dBm, and the general noise is −70 dBm. Thus, an 8 dBm power attenuation results from passing through the metal case.

This outcome reveals that the strength of the signal in air ranges from −70 dBm to −12 dBm, indicating that a power output of 58 dBm can be measured at a distance of 20 mm using the RF chip. However, the power declines by 13.8% after passing through the metal case, though it remains within the resolvable range of the chip. Therefore, the RF chip-powered signal transmission module is capable of transmitting signals normally through metal cases.

### 3.2. Framework of Diagnosing Three Fault Types

The WPMS is designed to replace traditional fault diagnosis systems. The traditional measurements lead to structural changes in the spindle system, and the signal cables cannot be placed inside the spindle. Therefore, most monitoring systems attach the accelerometer outside the case of the spindle to monitor vibrations. The WPMS developed in this study can be placed inside the spindle, and uses the shear force position of the bearing closest to the spindle to measure vibrations of the spindle system, and determine the cause of the vibration. This study purposefully creates three typical faults in the spindle, namely, the unmatched assembly dimensions of the spindle system, an unbalanced system, and bearing damage. The results of monitoring vibrations from inside using the WPMS and from outside using an external monitoring system are compared.

**Figure 5.**
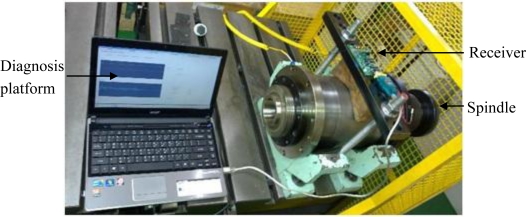
The testing platform of the PWFDS.

First, a testing platform that uses a motor to drive the spindle to spin is constructed. Revolutions of the motor drive the embedded power generator hidden in the spindle, and the WPMS transmits vibration data packets to the outside of the spindle to be read on a computer at the receiving end through a RS-232 interface. The structure of the system is shown in Figure 5.

This study simulates the three most frequently occurring causes of spindle faults, the unmatched assembly dimensions of the spindle system, an unbalanced system, and bearing damage, and these causes are described as follows:

#### Case 1: Unmatched assembly dimensions of the spindle system

The fault of unmatched assembly dimensions of the spindle system can be attributed to the installation of a spacer ring, where the dimension error of the spacer ring in the spindle is greater than what is allowed. This fault typically occurs due to human error during spindle production, inaccurate measuring devices, and excessive temperature difference. Therefore, during the running-in tests, the spectrum plots of the spindle vibrations are measured from a slow revolving speed of 1,000 rpm to as rapid as 5,000 rpm.

#### Case 2: An unbalanced spindle

The fault of an unbalanced spindle occurs during the assembly of the spindle or during the operation processes. If left unresolved, this fault can lead to the production of imprecise products, overheating of the spindle, or bearing damage over a long duration. The fault was simulated by attaching a screw with a weight of 5 g to the joint between the rotor and the connecting rod to create an unbalanced force. During the running-in tests of the spindle, the spectrum plots of the spindle vibrations were measured from a slow revolving speed of 1,000 rpm to as rapid as 5,000 rpm.

#### Case 3: Damages to the bearing retainer

This fault often occurs in deteriorated or destroyed spindles that have been repaired numerous times. If left unresolved, the fault may lead to an overheated or destroyed spindle over time. During the initial stage, this fault can only be detected in the spectrum graph of the monitored vibrations. The bearing used in this experiment was dilapidated, replaced during production, and then used with the WPMS for monitoring.

All three types of fault are summarized in Table 3.

### 3.3. Actual Application on the Production Line

**Figure 6.**
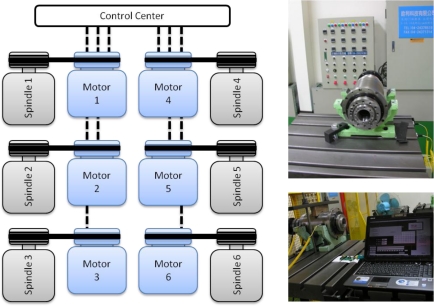
The running-in test system in the spindle production line.

The WPMS is used in the spindle production line to monitor whether any defects exist in the assembly process of the spindle. The system can also be used as a factory certification, maintaining a record of the vibration history in spindle production. Following assembly, each spindle must undergo a running-in test to verify that the spindle can operate from low to maximum speeds. Another objective of this running-in system is to evenly lubricate the bearing of the spindle system. Temperature changes are monitored constantly during the production process. The spindle is tested on this platform from a low speed of 1,000 rpm to a high speed of 5,000 rpm. In this study, the WPMS underwent a running-in test in the spindle production line, as shown in Figure 6. This experiment monitors whether the WPMS experiences interference when the spindles are under normal conditions, and when all the other spindles are experiencing serious faults in the testing platform.

## 4. Results and Discussion

The primary purpose of the WPMS is to monitor whether any faults occur during the operation of the spindle. This study simulates the three most frequently occurring causes for spindle faults, namely, the unmatched assembly dimensions of the spindle system, an unbalanced system, and bearing damage. Through these three fault types, differences between monitoring fault vibration signals across metal cases using a WPMS and that of using traditional methods are compared. The result of the tests can be used to analyze the fault identification rate, and to address practical problems in the production line of spindles.

**Figure 7.**
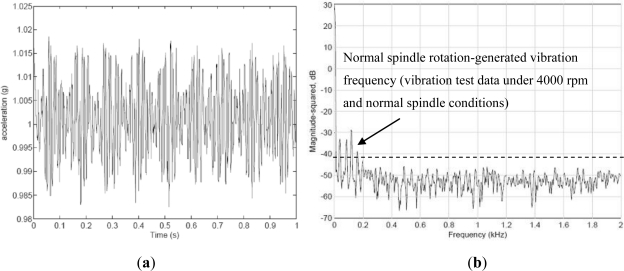
The time domain and spectrum of vibrations when the spindle revolves at 4,000 rpm. (**a**) Data in the time domain. (**b**) Spectrum of the vibrations.

In this study, the revolving speed of the spindle is maintained constant at 4,000 rpm, because faults are more easily identified when operating over 4,000 rpm. The time domain of vibrations when the WPMS monitors the vibration of the spindle under normal conditions is shown in Figure 7(b). Because the cause of spindle faults are not easily analyzed through vibration signals in the time domain, data sampled at a frequency of 0.25 ms/time were transferred to a frequency domain using Fast Fourier Transforms (FFT), and the number of sampling points was set at 1,024. The resulting frequency domain is shown in Figure 7(b). The spindle of the machine tool used in this study generates a vibration frequency of 180 Hz under normal conditions and at a speed of 4,000 rpm, which is a resonance generated by the revolving spindle. Under different revolving speeds, the vibration increases as the revolving speed increases. Other low frequencies result from the low-frequency vibration generated by the motor of the testing platform. A normally functioning spindle system should not produce any high-frequency vibrations apart from the primary vibration frequency generated during normal operations.

### 4.1. Result of the Experiment on Case 1, where Deviation of the Parts of the Spindle is Excessive

**Figure 8.**
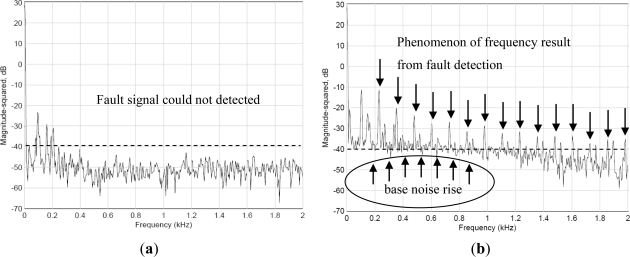
(**a**) The spectrum obtained using traditional methods, where no vibrations caused by faults were detected. (**b**) The multiplied vibration frequencies obtained using the WPMS when faults occur.

In this experiment, a component was installed inside the spindle with a spacer ring that had a size tolerance greater than the allowed value. Spectrum data were monitored using the WPMS when the spindle was in operation. The data was then compared with the spectrum obtained from monitoring the exterior of the spindle. Because of the incorrect assembly tolerance, the non-linear reaction of the loose component acting on the spindle caused numerous harmonic vibrations, leading to truncated vibration waveforms and a hoisted noise floor in the spectrum. When this fault type occurs, the phases are typically unstable, each measurement possesses a different result, and the revolving shaft changes position as the assembly becomes loose. Therefore, the value of vibrations measured by the same bearing will continually change when the measuring positions change. Loosened components frequently lead to sub-harmonic vibrations on the spectrum. Figure 8(a) depicts the vibration spectrum obtained through traditional methods, where vibration sensors are mounted on the outside of the case. The results show that no abnormal vibrations caused by the loose component within the spindle were observed. In contrast, the vibration signals monitored using the WPMS, as shown in Figure 8(b), were displayed on the signal spectrum immediately when a fault occurred because of the harmonic vibrations caused by a loose component. A comparison between the two experiments revealed that the innovative WPMS can detect abnormal vibrations more effectively when a fault occurs.

### 4.2. Result of the Experiment on Case 2 with an Unbalanced Spindle

This experiment identified the imbalance of the spindle system using the wireless signaling module, and compared the result with the vibration signals obtained from the outside of the case. In this experiment, a screw weighing 5 g was attached to the rotor shaft, creating an imbalance in the spindle system. Generally, vibrations caused by unbalanced forces amplify the vibration amplitudes in correspondence to the higher amplitudes measured on the main frequencies.

**Figure 9.**
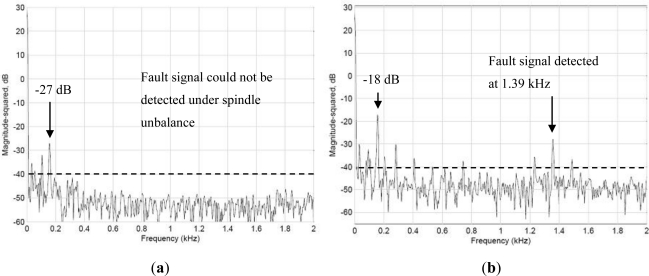
(**a**) The spectrum obtained using the traditional method, where no vibrations caused by faults were detected. (**b**) The 1.39 kHz vibration frequency obtained using the WPMS when faults occur.

Figure 9 shows the vibration spectrum detected when the spindle revolves at 4,000 rpm. Figure 9(a) shows the signals of the spindle detected outside the spindle case, and Figure 9(b) shows the vibration signals detected by the WPMS. A comparison between the two outcomes revealed that the vibration power detected outside the case using the traditional method was −27 dB, whereas that detected using the WPMS was −18 dB. Clearly, when an imbalance occurs in the spindle system, the amplitudes of the vibration increase, increasing the power of the main vibration frequency. Additionally, fault signals emitted at 1.39 kHz are also detected using the WPMS, which is the secondary cause for faults resulting from poor concentric bearing movements because of the system imbalance.

### 4.3. Case 3: Damaged Bearings on the Spindle

**Figure 10.**
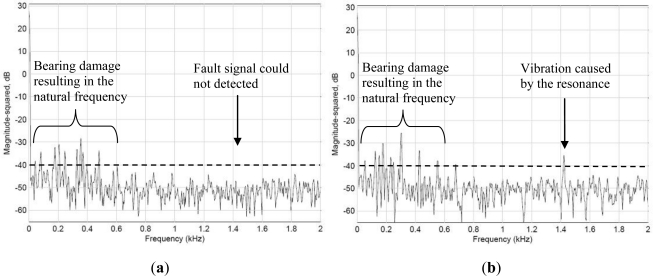
(**a**) The spectrum measured from the outside of the spindle using the traditional method. (**b**) The spectrum measured using the WPMS.

The objective of the system is to identify slight damage to the bearings. The primary fault frequencies range from 0 to 2 kHz. However, the more severely the bearing is deteriorated, the more damaging the frequency bands will become, and the total energy increases accordingly. Generally, when this occurs, the bearings must be replaced immediately to avoid deterioration from continuing. This experiment also simulates when a bearing is broken. The WPMS was used to monitor the vibration; the result is compared with the vibration signals obtained from outside the spindle case. Figure 10 shows (a) the fault signals detected by the WPMS, and (b) the fault signals detected outside the spindle case. A comparison between the two graphs in Figure 10 reveals that faults occurring on the bearing of the spindle frequently denote serious damage. Therefore, vibrations above 180 Hz can be detected from both inside and outside the spindle; nevertheless, greater vibration signals can be parsed from inside the spindle.

**Figure 11.**
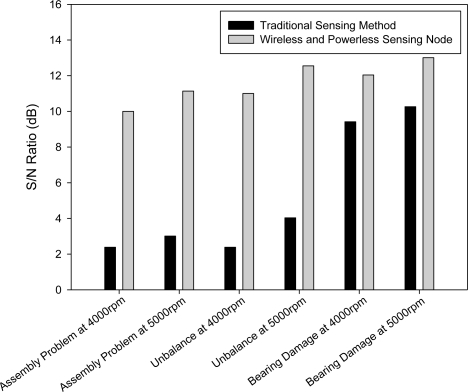
The WPMS possesses higher SNR compared to the traditional diagnosis method.

Figure 11 shows comparisons of the SNR of the signals in the three simulated fault experiments using the WPMS and external measuring. Regarding measuring system faults externally, the results indicate that, when applied to fault identification in Case 1 and Case 2, the signals were relatively weak, ranging from 2.4 dB to 4 dB; whereas when measuring Case 3, the SNR reached 10.3 dB. Therefore, when the traditional method is used to diagnose faults, the SNR of different system faults can vary significantly. When the WPMS is used to diagnose system faults, the SNR of the three simulations was between 10 to 13 dB. Moreover, the SNR was relatively consistent under all three fault testing conditions.

**Figure 12.**
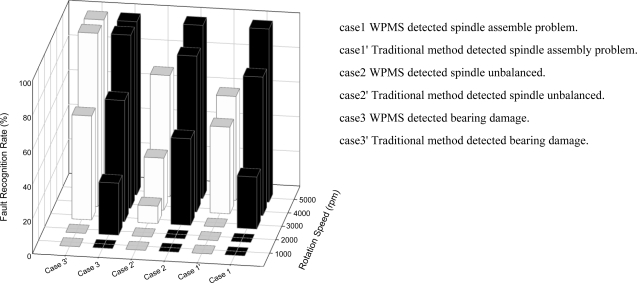
The WPMS provides a higher fault recognition rate than the traditional method does.

The WPMS discussed in this study was implemented in the testing and monitoring platform of a spindle production line. By conducting tests when the spindle revolved at various speeds between 1,000 to 5,000 rpm through a running-in system, the WPMS and the traditional fault monitoring system were examined extensively. Figure 12 displays the two methods of fault diagnosis, and each method was used on 10 spindle systems for fault identification. The results of Figure 12 indicate that greater revolving speeds render higher identification rates when used to monitor whether the spindle system operates normally, and that the WPMS has a higher identification rate than the traditional fault diagnosis method when revolving at a low speed. Under the same speed of 4,000 rpm, the minimum identification rate is 30% using the traditional method, whereas the minimum identification rate is 80% using the WPMS. Moreover, 100% of system faults are identified using the WPMS in the running-in system at the production line when the spindle revolves for a complete range from 1,000 to 5,000 rpm. This result indicates that the WPMS applied in the production line for fault recognition provides higher recognition rates.

### 4.4. The Results of Internal Sensing and External Sensing in Embedded Systems

**Figure 13.**
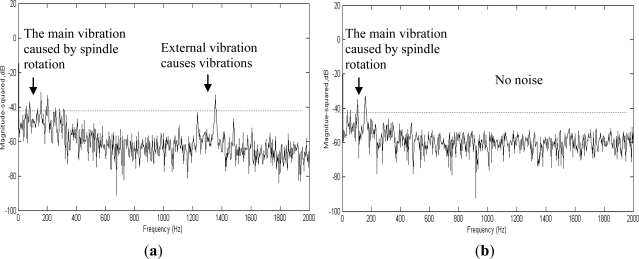
(**a**) Spectrums obtained externally experience interference from other spindle operations. (**b**) The WPMS is less likely to experience interference from other spindle operations.

In practical operations, either on machine tools or in production lines, the spindle functions with other systems. Therefore, if a hidden monitoring system is applied to ordinary monitoring tasks, the system must be clear of interference from other systems. This study detects the spectra inside and outside the spindle during the production of the spindle. Additionally, further analysis was conducted; the test employed six spindle testing platforms in a factory, which were operated simultaneously. A faulty spindle system was placed at the Number 2 spindle to monitor the operational data from the Number 6 spindle to determine whether the hidden fault diagnosis system will experience interference when applied to operational monitoring. The results of the experiment are shown in Figure 13; Figure 13(a) displays the spectrum of the hidden fault diagnosis system, whereas Figure 13(b) displays the spectrum monitored externally. The results show that during internal monitoring, the primary vibration frequency occurred only at 80 Hz when the spindle revolved at a speed of 4,000 rpm. However, the spectrum observed externally suggests that the spectrum experiences interference from other operations at 690 Hz, and significant interference was experienced at the primary vibration frequency of 80 Hz. Therefore, using a diagnosis system hidden inside the spindle for spindle faults, not only enables the monitoring of faults in the spindle system, but also prevents external interference. Thus, health condition inspections and monitoring can be conducted either during spindle production or machine tool production.

## 5. Conclusions

This paper extends the research reported in the journal article titled *Wireless and Powerless Sensing Node System* (*WPSN)* [11]. We developed a key technique that mounts a Wireless and Powerless Monitoring System (WPMS) inside a spindle, and transmits signals through a metal case. After lengthy tests and adjustments, the WPMS was successfully used to monitor the spindle system. Under the wireless framework, the MEMS accelerometer was incorporated to monitor the vibration signals of the spindle.

The three most commonly occurring faults are unmatched assembly dimensions of the spindle system, an unbalanced spindle system, and bearing damage. Comparing the WPMS with the traditional external fault diagnosis system, the experiment results showed that the WPMS provides superior Signal/Noise Ratio (SNR) and provides better fault-signal characteristics. Implementing the WPMS inside the core of the spindle enables vibration detection when a fault occurs. The results of the experiments verified that the fault recognition rate of the WPMS is 62.5% higher than that of the traditional method when the spindle revolves at 4,000 rpm; higher speeds result in better recognition rates. The WPMS provides better resolution capabilities compared to traditional monitoring methods.

Furthermore, the experimental results demonstrate that the WPMS was not affected by signals generated by other spindle vibrations when applied to the spindle production line. By installing a machine tool, system abnormalities can be detected effectively to prevent shutdowns without prior warning. Reducing unexpected system shutdowns ensures greater production stability at a factory and also reduces operating costs. Therefore, a spindle equipped with a WPMS will be a valuable testing technology for factories in the future.

## Figures and Tables

**Table 1.** Accelerometer specifications [12,13].

**Parameters**	**Value (MEMS)**	**Standard industrial acceleration sensor**
Range	±6 g	80 g
Operating temperature range	−40–85 °C	−50–120
Sensitivity	819 counts/g	100 mV/g
Sensitivity variation from RT over temp	−2–+2%	±5%
Resolution	1.22 mg	200 μg
Non-linearity	1%	≤1
Noise density (on filter pins)	175 μg/√Hz	20 μg/√Hz
Cost	≒ 50 USD	≒ 300 USD

**Table 2.** nRF905 signal transceiver chip specifications [14].

**Parameter**	**Unit**	**Value**
Supply voltage	V	1.9–3.6
Maximum transmit output power	dBm	10
Transmitted data rate	kbps	100
Supply current in transmit @ −10 dBm output power	mA	9
Supply current in receive mode	mA	12.5
Operating temperature range	°C	−40 to +85
Typical sensitivity	dBm	−100

**Table 3.** Simulation of fault occurrence.

Case 1. The excessive dimension error of the spindle parts	**Implementation:** During spindle production, a part with the incorrect dimensions was assembled at the spindle.
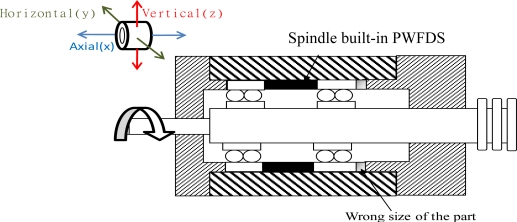	
Case 2. Unbalanced spindle	**Implementation:** Attach a 5 g screw to the rotor of the spindle to create an imbalance in the spindle system.
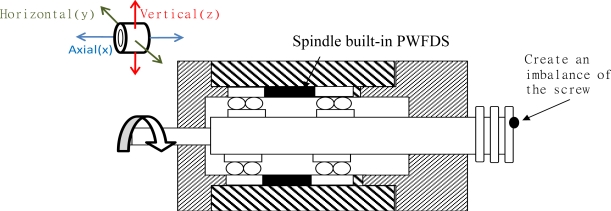	
Case 3. Damage to the bearing of the spindle	**Implementation:** Place a abrasion bearing retainer that presents a workable bearing inside the spindle where embedded fault diagnosis is installed.